# The Tragedy of Liberal Democratic Governance in the Face of Global Threats

**DOI:** 10.3389/fpubh.2022.902724

**Published:** 2022-07-08

**Authors:** Eric Muraille, Philippe Naccache, Julien Pillot

**Affiliations:** ^1^Laboratoire de Parasitologie, ULB Center for Research in Immunology (U-CRI), Université Libre de Bruxelles, Gosselies, Belgium; ^2^INSEEC Grande Ecole, OMNES Education, Paris, France

**Keywords:** global threats, COVID-19, liberalism, One Health (OH) approach, neutrality of the state, individualism and collectivism, postmodernism

## Abstract

In hindsight, the early response of liberal governments to the SARS-CoV-2 pandemic was chaotic and generally inefficient. Though one might be tempted to attribute these failures to the incompetence of certain political decision-makers, we propose another explanation. Global threats require a coordinated international response, which is only possible if the threat is perceived in the same way by all, and if government priorities are similar. The effectiveness of the response also relies on massive adhesion of citizens to the measures imposed, which in turn requires trust in government. Our hypothesis is that certain fundamental features of liberalism complicate such global and collective responses: neutrality of the state and primacy of the individual over collective society. Liberalism considers that institutions and public policy must not be designed to favor any specific conception of the common good. That which is best for all is usually determined by a “competition of opinions,” which frequently leads to scientific expertise being considered as only one opinion among many. Liberalism also imposes strict respect for individual freedoms and private interests and tends to reject any form of collectivism or dictate imposed by the common good. In order to solve these structural problems and improve society's management of global threats, we make several proposals, such as the introduction of a minimal and consensual definition of the common good and the promotion of a health policy guided by One Health-like concepts. Overall, our analysis suggests that because political ideologies provide their own definitions of the common good and the place of scientific knowledge in the governance process and can thus affect the response to global threats, they should be urgently taken into consideration by public health experts.

## Introduction

Between, November 2019 and the appearance of the Severe Acute Respiratory Syndrome CoronaVirus 2 (SARS-CoV-2) responsible for Coronavirus disease 2019 (COVID-19) in Wuhan, China (1) and December 2020 when the Pfizer/BioNtech vaccine was authorized by the US Food and Drug Administration (FDA), the only measures available to governments to limit the spread of the virus were testing, tracing, isolation and requiring mask-wearing and social distancing, as recommended by the World Health Organization (WHO). Retrospective analyzes (2) estimate the number of people who died of Covid-19 during 2020 at 18.2 million. However, these deaths are very unevenly distributed among countries. The highest levels of excess mortality were observed in the USA, Brazil, Mexico, India, Pakistan, Russia and Indonesia. But some countries, such as New Zealand, Singapore, South Korea, Taiwan (2) and even China (at the exception of Wuhan) (3) succeeded to control the dissemination of SARS-CoV-2 on their territory and display very low and sometimes even negative excess mortality, which demonstrates that controlling the pandemic was indeed possible. Consequently, we can consider that all countries that have been unable to prevent the spread of SARS-CoV-2 and a significant excess mortality have failed to deploy an effective health strategy, even if they have made meritorious efforts to achieve this goal. Our main objective is to try to understand the reasons for this widespread failure of the liberal democracies which had significant economic, technical and scientific means and which also had for the most part the advantage of benefiting from a period of a few weeks before being confronted with the pandemic.

In retrospect, liberal democratic government responses to COVID-19 pandemic, which mainly required good organization and communication with citizens, often came too late to prevent the spread of the virus and above all appeared very disorderly. Each government implemented its own strategy with little international coordination, which sometimes led to absurd situations. For example, some European countries opted at the start of the pandemic for a containment strategy, while certain neighbors, like the Netherlands (4) and the United-Kingdom (5), adopted a “*laissez-faire*” strategy in the hopes of quickly obtaining natural collective immunity. This lack of coordination was even observed between regions or states of the same country. For example, in the USA, each state pursued its own virus containment measures, regardless of what its neighbors were doing, which proved particularly counterproductive (6). The Covid-19 crisis has also been characterized by an anti-science attitude of several political leaders (7–10) who publicly denied the danger of the epidemic, the effectiveness of social distancing measures or even promoted unvalidated therapies. This generated strong political divisions and reduced the acceptance by citizens of public health measures. These governance failures led editors of reputable scientific journals, such as *The Lancet* (11), *Scientific American* (12) and *The New England Journal of Medicine* (13), to strongly condemn the political management of the Covid-19 pandemic in Europe and the USA.

The wide variations in the assessment of the danger as well as in the choice of the control strategies implemented cannot be attributed to a lack of scientific information, which has been widely available to all governments throughout the crisis. For example, the genome sequence of the SARS-CoV-2 virus was shared freely early, on 10 January 2020 (1), less than 2 weeks after the first cases were discovered in Wuhan City, which led to the WHO's publication on January 13 of the first diagnostic RT-PCR test protocol for identifying infected individuals. Information on the spread of the epidemic and on control methods was also shared in real time, by leading scientific journals and the WHO, which has been extensively involved in providing training and technical assistance on its OpenWHO platform and in deploying experts via its Global Outbreak Alert and Response Network (GOARN) (14).

Many scientists have blamed political leaders for their failure to control the epidemic, and hoped that democratic elections would solve the problem. This was surely behind the unprecedented decision of the editors of *The New England Journal of Medicine* (13) and *Scientific American* (12) to break with their century–old political neutrality to call for a vote against Donald Trump, who they accused of having undermined public confidence in science and public health institutions. However, the leadership failure hypothesis (HYP1) alone hardly accounts in full for the mismanagement by most liberal governments of the Covid-19 crisis, unless one accepts the idea of generalized incompetence of political decision-makers, which is an extreme position that lacks credibility. Furthermore, this mismanagement was unfortunately not an exception. Indeed, it even seems to be the rule in the face of global threats such as global warming, atmospheric and plastic pollution, or the disappearance of biodiversity. These threats and their potential consequences have been known for decades, and it is widely accepted that control policies have failed to prevent them. This led the scientific community to publish an editorial in October 2021 in 233 health journals calling for “*emergency action to limit global temperature increases, restore biodiversity, and protect health*” (15). What is the explanation for this tragic impotence?

We propose that, notwithstanding certain highly dysfunctional cases such as the Trump administration, the key to making sense of the recurrent tragic failings of liberal democratic governments in the face of global threats lies in certain structural governance features of democratic liberal political regimes themselves (HYP2). In this article we analyze and discuss this hypothesis. We will first define the key elements that are essential for an effective response to global threats and will then attempt to determine whether liberal ideology conflicts with these elements. Finally, we will suggest ways to improve governance in the face of global threats.

### Global Threats Require Science-Driven Global Responses

The exceptional advances in science and technology over the 20th century have had multiple consequences. We see two major ones as being linked to global threats. First, the large-scale and uncontrolled deployment of modern technologies have dramatically increased the risk of extinction of the human species as well as the nature of the threats it faces. Today, the probabilities of anthropogenic extinction linked to technologies (war, climate change, pollution, etc.) outweigh the causes of natural extinction, such as asteroid impacts and super-volcanic eruptions (16). Second, modern technologies have allowed for the emergence of deep social and economic interconnectedness between human societies. The advent of this “global village” has had many advantages but has also globalized the risks associated with epidemics. Infectious agents spread with alarming speed (17). In 2003, SARS-CoV-1 reached Toronto (Canada) from Hong Kong in only 24 h thanks to tourists (18). In 2009, the WHO declared H1N1 a pandemic (19) less than 3 months after it was first detected. In 2020, SARS-CoV2 was also declared a pandemic after only 3 months (1). This rapidity of dissemination has had profound consequences. We cannot hope to develop, validate, produce and distribute vaccines in just months to fight every infection at the planetary level. We are only left with social distancing measures to deal with these epidemics, which engenders significant human and economic costs (20, 21).

**Figure 1:**
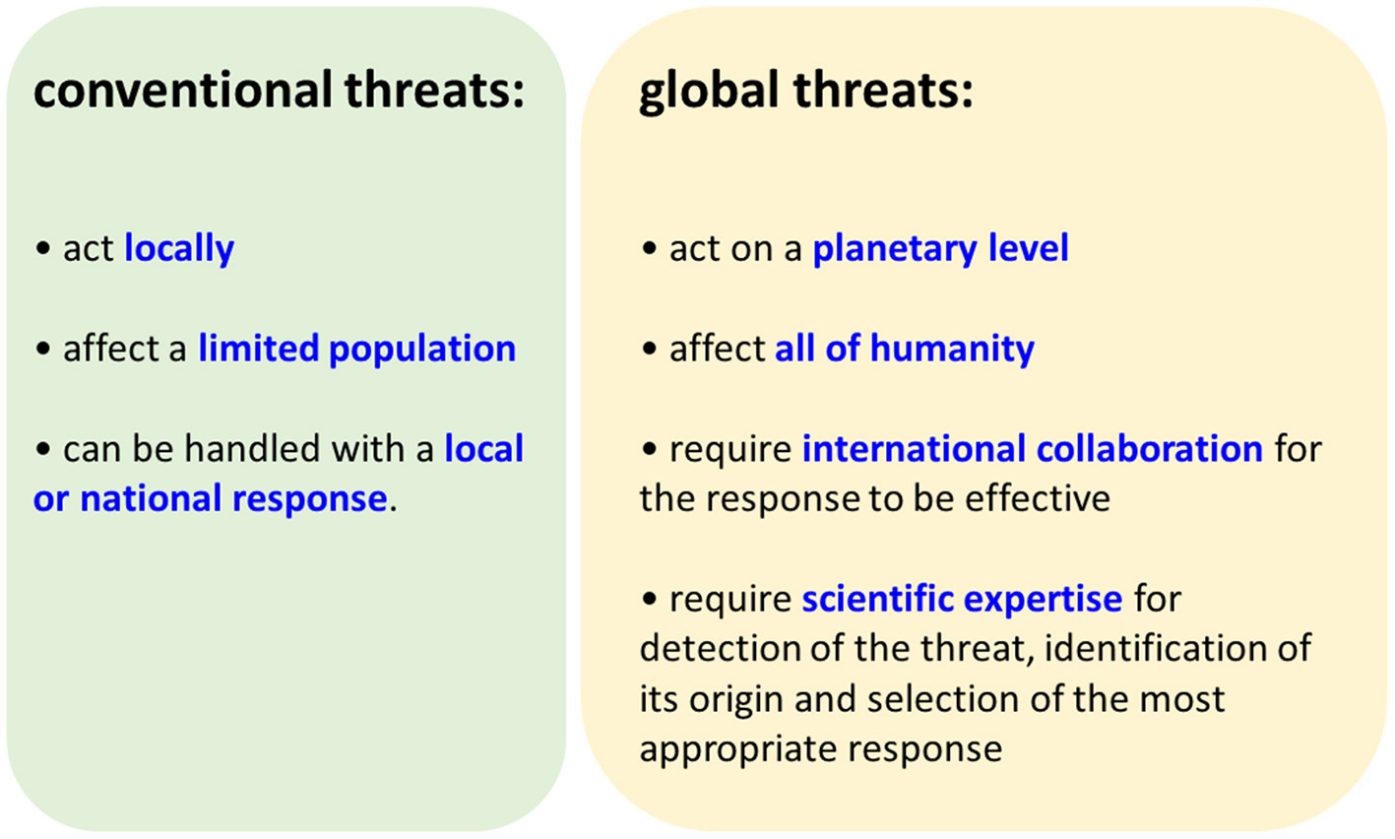
Comparaison of conventional and global threats.

**Figure 2:**
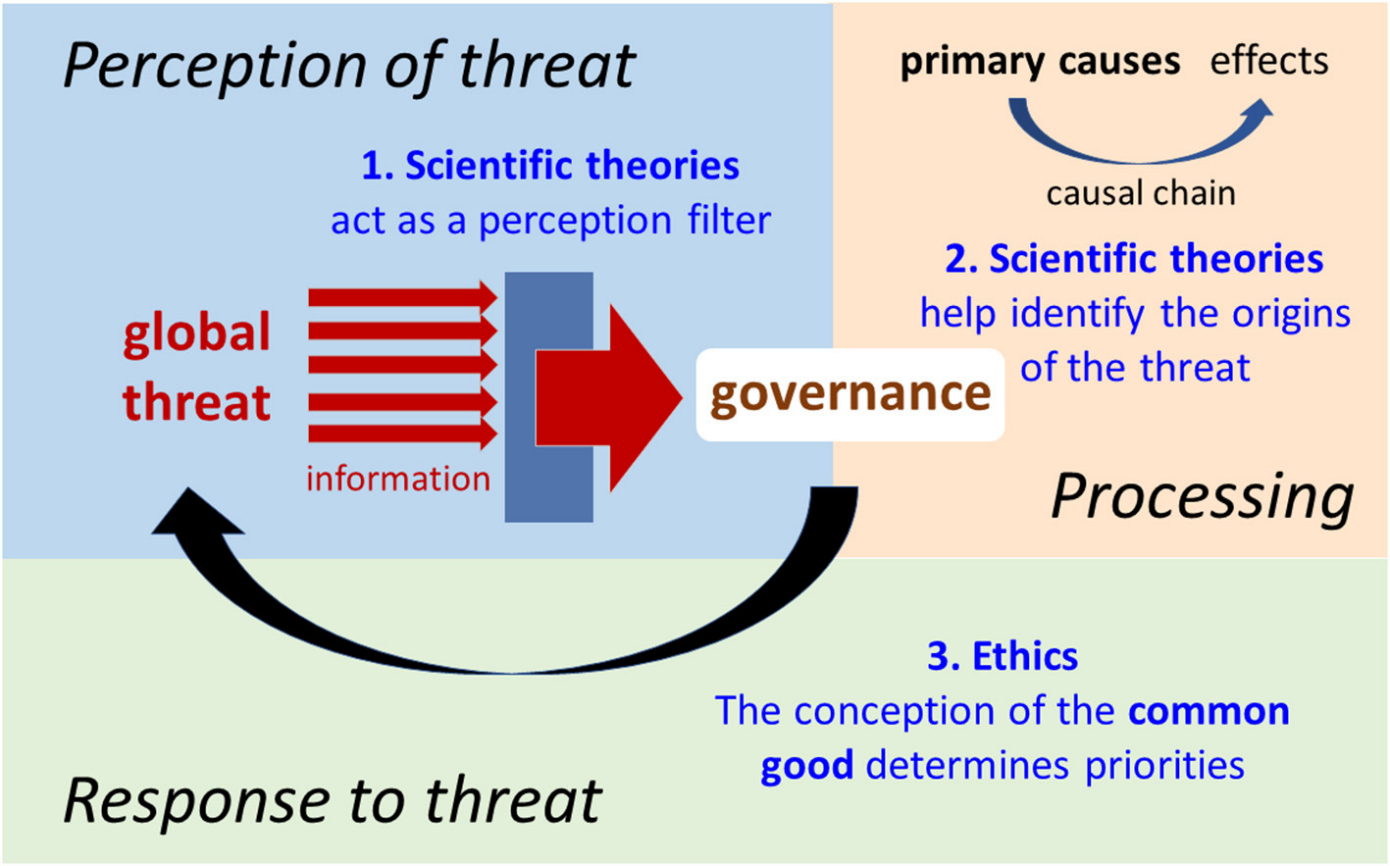
Schematic view of the decision-making process in the face of global threats.

We can conclude that modern technologies have allowed for the emergence of a new categories of threats, “global threats” (i.e. able to negatively affect the entire human species). These threats differ fundamentally from conventional threats in several areas, and consequently require specific responses (Figure 1). They all have in common the fact that they cannot be dealt without a coordinated international (global) response. In practice, this implies a large consensus on both the nature of the threat and the priorities of the response required (Figure 2).

Common sense is of no use when interpreting epidemiological or climatic data. Scientific theories are indispensable for detecting global threats, predicting their consequences, identifying their origins and finding ways to deal with them. We propose one simple but striking historic example to illustrate this. When they were discovered in the 1930's, chlorofluorocarbons (CFCs) were considered a miracle product. They were non-toxic and relatively inexpensive to produce (22). However, in the early 1970's, two chemists, Molina and Rowland, discovered that CFCs persist in the atmosphere and their derivatives, free chlorine atoms, cause a significant reduction in the stratospheric ozone layer that protects us from ultraviolet irradiation (23). In 1985, the meteorologists Gardiner and Shanklin and the geophysicist Farman discovered the Antarctic ozone hole and predicted a gradual decline in stratospheric ozone levels over the long term (24). These scientific discoveries, and their confirmation by other studies, led to the 1987 Montreal Protocol that totally banned all CFCs, and to the establishment of a multilateral fund to provide financial assistance to help developing countries abandon the use of CFCs. This example shows that science has a unique and irreplaceable role to play in the detection, prevention and response to global threats. Without it, the threat posed by CFCs would never have been identified and neutralized. The assessment of the consequences of disappearance of the ozone layer also required a multidisciplinary analysis. Convincing the CFC-producing countries to stop producing them required universally acceptable arguments, that is to say ones that were rational and based on verifiable empirical facts.

Addressing global threats effectively also requires a consensus on the priorities in the response in order to achieve concerted action at both the national and international level. And this requires a clear, shared definition of the common good, which is far from the case now. During the COVID-19 crisis, for example, we saw many governments hesitate between protecting the economy, individual freedoms, religious practices or health. This question of priorities arose throughout the crisis and disrupted its management.

We can conclude that the management of global threats questions both the value of scientific knowledge as well as the very meaning of the common good. In the remainder of this article, we analyze how liberal ideology and democratic governance answer these fundamental questions. As the very nature of liberalism itself is the subject of intense debate, and in order to avoid misunderstandings, we include a brief historical perspective of what we consider to be its main features.

### Neutrality of the Liberal State and its Consequences on Governance

Liberalism emerged in the 17th century and gradually came to dominate in Western nations by the 18th century, in a world of increasing interconnectedness and international trade thanks to scientific progress but which remained deeply divided and ravaged by wars of religion. Consequently, its main concern was the pacification of society. To this end, the pioneers of liberalism proposed a major break with all previous socioeconomic systems. While the legitimacy of governance during antiquity and the Middle Ages derived from religious morals and therefore from a codified representation of the good, liberalism proposed to clearly dissociate political governance and morality and reject all religious or philosophical normative systems. As heirs to the Enlightenment and inspired by the successes of physical sciences, the pioneers of liberalism attempted to replace divine laws with natural laws and introduce a mechanistic view of human nature in order to legitimize political decisions.

Already in 1748, the French philosopher Montesquieu put forward that “*Peace is the natural effect of trade. Two nations who traffic with each other become reciprocally dependent*” (25). Later, in 1758, in *De l'esprit*, the French philosopher Claude-Adrien Helvétius proposed that “*If the physical universe is subject to the laws of motion, the moral universe is no less subject to those of interest*” (26). These ideas would later greatly influence pioneers of philosophical and economic liberalism such as Jeremy Bentham, Adam Smith and John Stuart Mill. The free pursuit of private interest through trade is the natural engine of the economy and consequently must constitute the self-organizing principle of governance. This is what the Scottish philosopher and economist Adam Smith theorized in 1776 in *An Inquiry into the Nature and Causes of the Wealth of Nations* (27), considered one of the founding works of liberal economic theory.

Thus, although often regarded as such, liberalism is not axiomatically neutral. One of its fundamental traits is the belief in the ability of competition to auto-organize and optimize economic, social and decision-making processes. This legitimizes the “*laissez-faire*” of free economic markets as well as the process of deliberative democracy which subjects different societal projects to the evaluation of the public or of a representative assembly.

This faith in competition as an auto-organizing principle implies conferring on all a large degree of individual liberty and an equal right to participate in the decision-making process, regardless of their sense of the common good. Thus, according to the American philosopher John Rawls (28), a liberal democratic state must show neutrality, in the sense that institutions and public policy are not to be designed to support or favor any one philosophical or religious conception of the common good over another. Along the same lines, the American philosopher Charles Larmore states that “*In a liberal political order, political principles are to be neutral with respect to controversial views of the good life*” (29). We should note, however, that for Larmore (30) there are “neutral values,” i.e., values like the economy, which are not controversial for a majority of citizens and on which the state can take a position. The state should therefore be neutral only on what is controversial.

Liberalism considers that people participate in the society through collective decisions in order to find good solutions (31). This collective process is achieved, according to Rawls (28), through the principles of free association in communities of interest in which people and groups – with equal liberty – pursue their own ends. The collective decision-making process takes the form of what we call a “competition of opinions,” in which each person and/or group has the possibility to claim and try to impose its own sense of the true and the good and formulate one or more principles of action. The Austrian economist Joseph Schumpeter defined liberal democracy as “*an institutional system leading to political decisions, in which individuals acquire the power to rule on these decisions at the end of a competitive struggle over the votes of the people*” (32). As a result, society becomes an area with a “pluralism of values” and those values are often incommensurable and mutually exclusive. Because there is no way to rank the values, “*a pluralism of values leads to a version of moral relativism*” (33).

In this context, truth tends to be decided by opinion polls, referendums or popular media. However, at the same time, what is considered as true is increasingly confused with what conforms to moral standards [cultural cognition of risk theory developed by Kahan et al. (34)] or that which is desirable by the greatest number. In addition, the most powerful community or group often distorts the deliberative process, especially with regard to public policy in the areas of health (35) and the environment (36).

By what precedes, we suggest that the following elements are the cornerstone of liberalism regardless of the different stream with this philosophical stance: (i) faith in the self-organizing capacity of competition (ii) defense of individual freedoms, (iii) the neutrality of the state concerning the definition of the common good. Indeed, these three traits are what distinguish all liberal governments from religious, communist or fascist governments. However, we emphasize that there are many debates about the interpretation, legitimization and possible implementation of liberal concepts. For example, Simon Caney (37) describes several conceptions of neutrality and numerous consequentialist type arguments legitimizing it. We did not want to introduce here these multiple interpretations. We have to make clear that our intention is not to write a paper about the philosophy of liberalism. We have a more modest scope which is to shed light on the relation between the most common traits of liberalism and public health policy which influenced the management of the COVID-19 pandemic.

### Evidence Based Medicine vs. Medical Populism: The Hydroxychloroquine Controversy

At this point, it is important to highlight the fact that the liberal process to determine what is true differs greatly from the process that science is supposed to use to produce true knowledge. In medicine, for example, the validity of a treatment is not supposed to be determined by simple referendum and even less by opinion polls.

The medical sciences have developed procedures that obviously exclude so-called authoritative arguments as well as those based on popular belief or desirability, in order to reach a consensus on what is true. These procedures, collectively referred to as Evidence Based Medicine (EBM), first appeared in the 1980's and were formalized starting in the 1990's (38). Since then, a hierarchization of the existing types of evidence has gradually come to be accepted as the norm of EBM (39). Expert opinions are considered to have the highest level of bias and be the least reliable. Then come case studies, studies on small cohorts of individuals (a few dozen) and finally randomized studies on large groups of individuals (several thousand). In randomized studies, the validity of a drug, for example, is measured in a large number of people, with one group receiving the drug and the other receiving a placebo. The study is said to be “randomized, double-blind” when neither the individuals receiving nor giving the treatment know whether it is the drug or the placebo. Regardless of the number of individuals, the diversity of the cohort or the number of internal controls, no study is considered to be completely free of bias. Consequently, building a consensus frequently involves metanalyses that compare the different studies available and attempt to summarize them by evaluating the share of limitations and bias associated with each study (40).

The controversy surrounding hydroxychloroquine as a treatment for COVID-19 (41) has revealed that the EBM procedure to decide what is true, even though undeniably the norm in modern medicine, is far from being accepted among the public, policy-makers and even certain researchers. In February 2020, Didier Raoult, an internationally renowned French microbiologist (42), publicly promoted this treatment in the absence of peer-reviewed clinical studies and against the advice of the WHO. He categorically refuses to conduct a randomized controlled clinical trial to validate the treatment and presents himself as a researcher at odds with a scientific community dominated by “methodology maniacs.” While Raoult's attitude has been harshly criticized by the medical community, certain prominent political figures such as Presidents Trump, Bolsonaro and Macron (7), and even several researchers in the human sciences (43, 44), have defended his point of view and his revolt against the tyranny of methodology. To fully understand this controversy, it is necessary to briefly introduce the two concepts of the value of knowledge which currently coexist in Western universities and how they are compatible with liberalism.

### Two Concepts of the Value of Knowledge

Two philosophical visions, realism and postmodernism, regarding the value of knowledge are currently taught in universities, and they are irreconcilable in their radical form. These theories are far from strictly theoretical and of academic interest only, and have had an enormous influence on the governance of our societies. We will briefly present the main features of each, necessarily using simplified terms given the complexity of the subject.

Philosophical realism defends the existence of an external reality independent of our mind and representations and adopts a materialistic and mechanistic view of the world, seen as composed of stable elements linked by causal relationships. Realism attempts to produce “universal truths” about the nature of reality by rejecting religious, political, philosophical, ethnic and gender biases and analyzing the world on the basis of rationality, observation and experimentation. Realists believe in a clear demarcation between science and pseudoscience based on the Falsification Principle proposed by Karl Popper (45). This is the confrontation of the explanatory and predictive capacity of a theory with reality, which alone must determine its value. As pointed out by Robert K. Merton, “*The acceptance or rejection of claims entering the lists of science is not to depend on the personal or social attributes of their protagonist; his race, nationality, religion, class, and personal qualities are as such irrelevant*” (46). It is important to mention that realism is generally associated with the modern positive view of scientific knowledge, considered as a source of not only technical but also social progress.

Postmodern philosophy was first conceived in the 1960's, based mainly on the works of French philosophers such as Foucault, Deleuze and Derrida and was later popularized by Lyotard (47) who defined the postmodern condition as “*an incredulity toward metanarratives*.” Religions, political ideologies and science, which attempt to give a coherent account of the world and of life and legitimize the powers in place, are all metanarratives. Thus, postmodernism inextricably links ethics, politics and science, rejects claims of the objectivity of science and privileges “lived experience” over rational or empirical evidence. Postmodernism is based on constructivist and relativist ontology and epistemology. It views the world as fragmented, changing, indeterminate and strongly dependent on our conceptual representations. Scientific theories are considered mainly as social constructions and not as true descriptions of reality. Postmodernism claims that there are no universal truths but only “local truths” (i.e., valid within social groups). In its radical relativist form, postmodernism also denies any possibility of judging one theory on the basis of the concepts of another (Kuhn's incommensurability of paradigms) (48). Consequently, postmodern philosophy rejects the existence of a strict demarcation between science and pseudoscience.

From a sociological perspective, as pointed out by the French philosopher Michel Foucault (49), metanarratives control what can be “known” by individuals and are themselves controlled by dominant institutions. Thus, people are defined by their social position and science is therefore above all a tool of dominance whose form and content are socially determined. For example, the feminist philosopher Sandra Harding claimed (50) that because modern science was mostly produced by straight white men, it is “*not only sexist, but also racist, classist and culturally coercive*,” and that “*physics and chemistry, mathematics and logic, bear the fingerprints of their distinctive cultural creators, no less than do anthropology and history*.”

For postmodernism, social progress and freedom are necessarily achieved through criticism and relativization of so-called universal scientific knowledge, which is considered to be an instrument of oppression. Richard Rorty, arguably America's most famous postmodern philosopher, claimed that the primary goal of liberalism should be to reduce cruelty, pain and conflict. According to him, most acts of cruelty stem from certainty and rationalization, which he alleges allow society to consider some people as less human, thus legitimizing cruelty. From a historical perspective, Rorty describes Enlightenment scientism as a holdover from the religious need to have human projects backed by a non-human authority. However, according to this philosophy, though rationalism was essential in the early days of liberal democracy, it now has come to hamper the preservation and progress of democratic societies. Rorty hopes for the advent of a pragmatic liberal society rejecting rationalism and asserts that “*a liberal society is one which is content to call “true” (or “right” or “just”) whatever the outcome of undistorted communication happens to be, whatever view wins in a free and open encounter*” (51).

### Postmodern and Liberal Views of Knowledge Converge and Legitimize Each Other

The postmodern idea that knowledge is produced in a context of application, that it is not neutral and does not require the independence of researchers is in perfect agreement with certain liberal-inspired economical models of knowledge production. On the other hand, the postmodern rejection of rationality, empirical evidences and the concept of truth also helps legitimize political communication based on emotionality and identity rather than rationality and facts. These two traits have strongly contributed to the diffusion and popularization of postmodern concepts among political decision-makers.

In 1995, Gibbons et al. (52) presented a theory based on the division of knowledge production into two main modes. “Mode 1” refers to the traditional linear mode of knowledge production in use since World War II: technological and organizational innovations are produced in universities and transferred to industry. The university is supposed to be an independent institution. In “mode 2” of knowledge production, innovations result from non-linear collaborative interactions between academia, industry and governments. Consequently, innovations are created in a context of application. The knowledge has a defined purpose, which fits well with the postmodern vision of knowledge serving the private interests of the dominant group.

In 2000, Henry Etzkowitz and Loet Leydesdorff proposed a general model for mode 2 production of knowledge, the “Triple Helix model” (53), based on interconnections between universities, governments and private companies. They insist that mode 1 knowledge production was only a transitory and unnatural state. The independence of universities was only necessary in order to protect them from Communist and Nazi ideologies. Then, Carayannis and Campbell (54) proposed to add a fourth major player to the Triple Helix, the “media-based and culture-based public and civil society,” thus giving birth to a “Quadruple Helix model.”

In the Triple and Quadruple Helix models, the highest possible number of stakeholders are expected to bring their specific expertise to the production of knowledge, which obviously presents undeniable advantages. However, the diversity of the motivations of the stakeholders also becomes the guarantee that the knowledge will meet the expectations of society. This form of democratic validation leads to the concept of “good science”: science that meets the expectations of society and respects social, cultural and ethical standards.

As discussed elsewhere (55), the epistemology associated with this good science widely deviates from Mertonian standards (46) and mode 1 realist epistemology, which attempts to produce universal knowledge by identifying and excluding bias and thus requires independence of the scientists working in universities. Conversely, mode 2 aims to produce useful science, corresponding to a societal demand, by mixing the expertise of universities with organizations whose interests, goals and methods differ widely. Good science is supposed to emerge not only from a confrontation with reality but also from the multiple interactions between the stakeholders. This vision of knowledge production is therefore again based on the liberal principle of the free market. Thus, competition is supposed to be the organizing mechanism of the economy and of governance, as well as of knowledge production and therefore of the perception of reality. We can therefore say that liberalism has gradually endowed itself with an epistemology, theorized by mode 2, the Triple/Quadruple Helix and postmodernism, in accord with its ontology. In the field of health, the liberal economist Milton Friedman suggested (56) that abolishing both medical licensure and the FDA in order to increase competition in the field of health care. This stance highlights the fact that, some of the most famous liberal theorists, can adhere to a postmodern view in matters of knowledge and to consider that the free market is able to select what which is effective in medical matters.

The communications of political leaders in Western societies have gradually entered a postmodern era (57). These last decades, political discourse has progressively evolved into a narrative (termed storytelling) based more on mythical and nostalgic stories and emotion than on objective facts and rationality. As summarized by Polletta et al. (58), “*Stories' persuasive power lies in their ability to call up other compelling stories*” but also in their ability to forge a collective identity. In this context, postmodernist concepts legitimize such political narratives, and “fake news” turns into an “alternative truth,” as characterized by Trump's campaign and presidency (59). In a postmodernist view, an alternative truth become the legitimate reality of one particular community and political discourse can break free from facts and rationality. This quickly turns out to be irreconcilable with science led by realism. It trains supporters of each political party to perceive reality in an increasingly biased way, which could partly explain the increased affective polarization (i.e., the tendency for members of a community or political party to dislike and distrust members of other communities or parties) within society observed during the Covid-19 pandemic (60). This polarization had major consequences on the acceptance of health measures. In the USA, wearing a mask was a sign of belonging to the Democratic Party and its rejection a Republican position (61). Nevertheless, this phenomenon is not limited to the USA, and has been documented in various societies (62).

Some authors, like Andrzej Szahaj (63), go so far as to speak of the advent of “postmodern liberalism” [which echoes the ideal of society defended by Rorty (51)] characterized by the absence of any inclination to claim to possess an ultimate truth. Postmodern liberalism therefore integrates constructivism by viewing all truth as ethnocentric, that is, as the product of a historical and cultural context. Any universalist proclamation must therefore be seen as an unacceptable form of coercion, except in situations where human rights are violated. It also abandons the legacy of classical Enlightenment humanism, for it avoids projecting any model of the human being at the level of perspective or the meaning of life. The reinforced neutrality of postmodern liberalism makes it viscerally opposed to scientifically established truths and thus to any science-driven policy.

It may be tempting to view the post-truth rhetoric of President Trump as a populist trait related to his personality and not as a postmodern form of liberalism. However, some authors, like Ernesto Laclau and Chantal Mouffe who are among the most prominent left-wing thinkers on populism, do not present populism as incompatible with liberalism. They consider, for example, that the policy adopted by the liberal Prime Minister Margaret Thatcher in the United Kingdom in the 1980s closely combines populism and liberalism: “*Stuart Hall has pointed out, for example, how Thatcherite populism combines the resonant themes of organic Toryism - nation, family, duty, authority, standards, traditionalism - with the aggressive themes of a revived neoliberalism - self-interest, competitive individualism, antistatism*” (64).

Since there is no consensual definition of liberalism and postmodernism. Our analysis of their convergence may not have unanimous support. However, our hypothesis has the merit of explaining several phenomena such as the increasingly frequent instrumentalization and delegitimization of scientific expertise by liberal political decision-makers, which we will now analyze.

### Liberal Governance Tends to Instrumentalize or Delegitimize Scientific Expertise

In 1959, the chemist and writer Charles P. Snow (65) already stressed that intellectualism in the whole of Western liberal society had become essentially divided into two cultures: the sciences and the humanities. He denounced the fact that most public and political decision-makers are ignorant of scientific advances and declared at a famous conference that “*the majority of the cleverest people in the Western world have about as much insight into it as their Neolithic ancestors would have had*.” In the same way, the psychologist Nathan S. Caplan noted that academic researchers and political policymakers “*live in separate worlds with different and often conflicting values, different reward systems, and different languages*” (the two communities theory) (66).

Thus, due to the lack of scientific knowledge within political bodies, but also because the pluralism of values within society is a cornerstone of liberal democracy, science frequently tends to become viewed as only one opinion among many in decision-making processes of liberal governments. As discussed previously, this attitude has been legitimized and reinforced by postmodern philosophy as well as by liberal theories of knowledge production such as the Triple Helix. In this context, liberal governments use scientific knowledge in a manner akin to the symbolic use described by Weiss (67). Lobbyists and politicians increasingly pressure scientific experts and science is frequently instrumentalized or silenced by politicians and business actors alike.

Organized disinformation campaigns and public denials of scientifically-established facts are not new. Such phenomena are common when science contradicts religious beliefs (68, 69) or conflicts with private commercial interests (70–75). However, they have taken on a new dimension during the Covid-19 crisis. Many leading liberal political leaders have publicly supported conspiracy theories and rejected the advice of official scientific experts (7–10). Certain organizations practicing disinformation have even started to actively collaborate and form powerful networks operating on a planetary level (76). Consequently, reports of personal attacks on scientists have been on the rise (77, 78). The proliferation of these practices excludes the possibility that they are simple accidents. On the contrary, we see the emergence of a new way of doing politics and communicating based on the promotion of alternative truths, which leads to fears that we are entering a “post-truth era” (79) characterized by public denials of scientifically-established facts and tolerance of politicians' lies, which would have tragic consequences in terms of public health.

As noted by *Science* magazine (80), President Trump long denied the danger of the Covid-19 pandemic and “*touted hydroxychloroquine as effective treatment and prevention for COVID-19 without evidence*.” He relayed videos on Twitter promoting a treatment whose effectiveness against COVID-19 had not been demonstrated (8). Trump and his administration also publicly attacked the credibility of several federal agencies like the Food and Drug Administration and the Centers for Disease Control and Prevention (81, 82). The consequences of this denial of the threat and discrediting of public health science were significant. Some estimate that it may have been responsible for 40% of the mortality due to Covid-19 in the USA (83).

Public denials of reality are unfortunately not limited to President Trump and have been observed among many liberal political leaders. In France, on March 6, 2020, President Macron ostensibly tried to reassure public opinion and protect the interests of the entertainment industry by appearing with his wife in the theater as the epidemic progressed in Europe (84). In England, Prime Minister Johnson initially refused to adopt containment measures and defended a policy of free circulation of the virus in the hopes of achieving collective immunity (5), before backing down in the face of the growing number of victims. In Brazil, President Bolsonaro continuously denied the danger of the epidemic and refused to implement any protective measures that could affect the economy (9, 10).

These anti-science attitudes clearly cannot continue to be seen as simply linked to the personality of certain political leaders or their scientific ignorance. The governments of countries such as the USA and England have access to the best expertise in public health. Trump's attitude, for example, cannot be seen as the result of simple ignorance, but as a deliberate will to lie and manipulate. As revealed by the journalist Bob Woodward, Trump was fully informed and aware of the dangerousness of the SARS-CoV-2 epidemic just as he was denying the risks (85). We propose that science denialism has become too frequent to be interpreted as accidental, and is instead the sign of a deep and very worrying structural problem linked to liberal governance.

### Science Denialism Leads to a Poor Perception of Reality and Weakens Epidemic Control

More than 100 years ago, *Science* magazine published an article on the lessons of the Spanish Flu pandemic (86). The first factor described as having hindered prevention was the inability of people to appreciate the risks involved. It seems that our societies have changed little since then. A quick comparison of the record of liberal governments in the face of the COVID-19 epidemic suffices to test the validity of our HYP2 and underscore the importance of science in the decision-making process.

**Figure 3:**
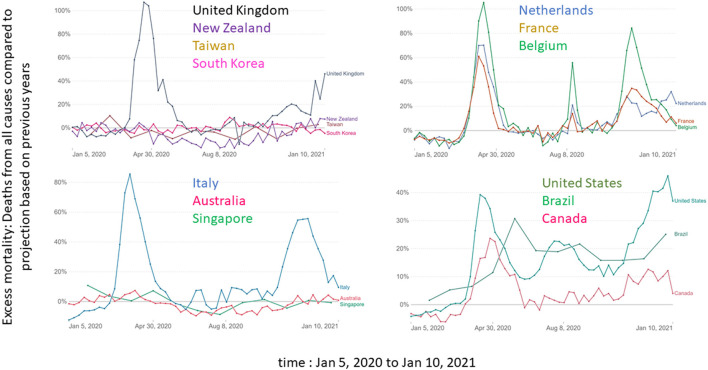
Comparison of excess mortality (Deaths from all causes compared to projection based on previous years) for Australia, Belgium, Brazil, Canada, France, Italy, Netherlands, New Zealand, South Korea, Taiwan, United Kingdom and United States between Jan 5, 2020 and Jan 10, 2021. The data comes from https://ourworldindata.org/.

From January 24, 2020, the WHO called for testing and isolation of travelers who may have been in contact with SARS-CoV-2 and to do everything possible to control the circulation of SARS-CoV-2. On March 16, 2020, the WHO Director-General declared on Twitter: “*the most effective way to prevent infections and save lives is breaking the chains of COVID-19 transmission*. *To do that, you must test and isolate*.” These simple recommendations, based on the available scientific data (87, 88) and past experience in the control of SARS-CoV-1 (89–91), were followed by very few governments. As a result, the number of people infected continued to grow exponentially and hospital systems quickly reached near full capacity in many countries. Only countries that anticipated or followed the WHO recommendations under the guidance of scientific experts, such as New Zealand (92, 93), South Korea (94) and Taiwan (95), were somewhat successful in controlling the spread of the virus (Figure 3). On the other hand, countries like the United Kingdom (5), USA (85) and Brazil (9, 10), whose governments publicly denied the threat and rejected or silenced scientific advice, saw the heaviest human toll. This suggests that the partial abandonment of state neutrality and seeing science as more than just opinion leads to better control of the epidemic. The fact that the USA was ranked first out of 195 countries in the 2019 Global Health Security Index, while New Zealand ranked 35, shows that adopting a science-led approach could be more important than the economic and technologic means available.

The abandonment of neutrality of the state and the adoption of a science-driven policy should not be confused with authoritarianism. Many notably authoritarian regimes, like Iran (ranked 151st out of 167 countries in The Economist's democracy index of 2019) and Turkey (ranked 110th), failed to effectively manage the Covid-19 epidemic (96) and some of the best performers were democracies, like New Zealand (ranked 4th), South Korea (ranked 23rd) and Taiwan (ranked 31st). Turkey reacted quickly and firmly to the epidemic, but made many decisions motivated by politics or religion, such as closing mosques late or allowing 350,000 people to attend prayer in Saint Sophia Cathedral in Istanbul. Iran's regime does not appear to have taken the pandemic seriously in its early stages and failed to adopt critical recommended public health protocols such as imposing domestic travel restrictions or enforcing social distancing measures. Moreover, when some measures were taken, they did not seem to have been applied correctly. Like Turkey, measures against COVID-19 in Iran were dominated by the religious and political concerns of the regime. For example, the Iranian government failed to quarantine the city of Qom, a holy site for Shiite Muslims, when the first cases of infection were reported there.

In summary, it is not enough to take authoritarian decisions to fight effectively against an epidemic. Retrospective analysis of the effectiveness of non-pharmaceutical measures in controlling the epidemic shows that some measures are more effective than others. Closing all educational institutions, limiting gatherings to 10 people or less, and closing face-to-face businesses each reduced transmission considerably (97, 98). However, there is no magic formula and a combination of measures must be taken and continuously adapted to local conditions (94). As these decisions are extremely painful for the economy and the population, they must be well understood and accepted by all, which requires both science-driven policy, trust in government and transparency, all things which are usually lacking in authoritarian regimes (96).

### The Failure of Individualism in the Face of COVID-19

Fighting a global threat requires large-scale cooperation, which can generate conflicts between short-term private interests and longer-term general interests. Consequently, the capacity of governments to motivate collective action and mobilize the entire population may have been a critical factor for successfully managing the COVID-19 pandemic. The behavior of individuals living in communities is regulated by moral norms and values. As discussed previously, faith in the self-organizing capacity of competition leads liberalism to promote individualism. This could constitute an additional structural weakness of liberalism in the face of global threats.

The divide between individualism and collectivism is a continuum based on the degree to which individuals see themselves as independent—vs. interdependent—of the society they live in. Western European and North American cultures dominated by liberalism endorse individualism, whereas most other cultures, such as those in Asia, share a stronger commitment to collectives such as country, tribe and family (99). While common sense suggests that the spread of the virus would be more intensive in collectivist societies due to their closer and more frequent social interactions, on the contrary it was collectivist societies that appeared to better control the pandemic (100, 101). One possible explanation for this may be the fact that a collective mindset favors sacrifice for the common good and adherence to COVID-19 health guidelines (100).

Energetic action by political leaders in favor of collectivist politics can play a decisive role, even in liberal countries. In New Zealand, for example, the government went to great lengths to not only inform and educate the public about coronaviruses but also to unify the population against COVID-19. Prime Minister Ardern frequently evoked “*a team of 5 million*” when calling for national unity and collective effort (93). This example illustrates well how a liberal democratic state can renounce its neutrality and invoke the collective interest in order to pursue a health policy. It also suggests that one of the priorities for politic leaders when faced with a major threat, in addition to adopting policies guided by science, should be to create a sense of shared social identity amongst the population that enables it to work together irrespective of individualistic or community feelings.

Of course, the importance of a good perception of the reality of the threat and of a collective response does not exclude the involvement of other factors such as demography, population density or experience acquired during previous epidemics such as the 2003 SARS to explain the varying capacity of countries to manage the COVID-19 pandemic. However, in practice, it is much easier for a government to adopt science-driven policies in a health emergency and unite the population in the face of a threat than to act on structural contingencies.

### The Fight Against Global Threats Requires a *Modus vivendi*

From the above, we can conclude that certain defining features of liberalism make it ill-suited to deal with global threats. Consequently, to successfully face global threats, we need to upgrade our decision-making process. We cannot just hope that a change in the composition of governments would solve the problem. This conclusion echoes a report from The Rockefeller Foundation-Lancet Commission: “*The present systems of governance and organization of human knowledge are inadequate to address the threats to planetary health*” (102). Hence, referring to the *modus vivendi* of Hobbes (103) which aimed to provide arrangements between members despite their differing interests, beliefs, values, ideologies, etc., we suggest that a minimum understanding be found of what the common good is and how to achieve it. This *modus vivendi* should be based on at least two arguments.

First, responding effectively to global threats implies convincing and uniting as many people as possible, regardless of their religious, political or social preferences. Thus, we propose that the survival of the human species as well as the preservation of human health must be seen as consensual ethical priorities for governance and embody the common good. This can be seen as an expansion of values considered as non-controversial and therefore neutral (in the sense defined by Larmore) for the state. Human survival and health are worthy and fair goals capable of convincing the greatest number since they are prerequisites for any other need or desire. Our “survival ethic” can be likened to Bentham's consequentialist ethic, in the sense that it is the consequences of our actions which determine their value. However, in the present case, the consequences at stake are those on the survival of our species and on health and not on our feelings of pleasure or pain, like in Bentham's ethic. The survival of our species may require difficult and painful choices, such as individual sacrifices for the benefit of the collective interest. Although it aims to be a minimal ethical basis, a simple *modus vivendi*, our survival ethic is also very different from the “minimal ethic” proposed by the French philosopher Ruwen Ogien (104), which can be reduced to one major principle: “*do not harm others, nothing more*” that is strongly liberal in inspiration. It aims to preserve maximum individual freedom and imposes neutrality with regard to justice and personal good. However, Ogien does not take into account the dependence of individuals on society for their survival and the interdependence of societies amongst themselves in an interconnected world. In addition, health cannot be considered only as a matter of individual choice because the health of one individual can have a strong collective impact, for example via the spread of epidemics. For example, in the case of certain infectious agents, it may be necessary to achieve vaccination coverage that significantly reduces the pathogen's capacity for dissemination in the population. This may involve making vaccination compulsory for the entire population.

Second, and related to the first argument, scientific advice must no longer be seen as just one opinion among others. Science is fallible, and does not produce absolute truths, but it is our most reliable method for comprehending natural phenomena and producing universal knowledge as a consensual basis for global decisions. Moreover, if a public health strategy becomes so politicized that it induces emotional polarization of the citizens toward it, support by scientists (non-politicized) for it tends to reduce the polarization and increase its acceptability (62). However, acting on the basis of science must obviously not lead to a single dogmatic vision of reality, nor to the systematic rejection of a plurality of methodological approaches to reality. We must reject the idea that knowledge is relative and that argumentation defines one's identity, as advocated by postmodern philosophy, as well as any proposal aimed at determining the reality of a fact by simple public referendum. However, though science reduces the uncertainty associated with facts and theories, it does not produce certainty. We must therefore find a way to reconcile scientific uncertainty with good governance.

### “One Health,” a Conceptual Scientific Framework for the Fight Against Global Threats

What conceptual scientific framework is compatible with a *modus vivendi* prioritizing survival of the human species and health, and would best allow us to prevent, anticipate and respond to global threats? One already exists and underpins the policy of most national and international public health agencies: the “One Health” concept developed based on knowledge accumulated in human medicine, veterinary medicine and ecology.

The “12 Manhattan Principles” were presented in 2004 in New York (USA), at a conference organized by the Wildlife Conservation Society (105). The first of these principles stresses the need to recognize the links between human health, animal health and the environment. Epidemics of zoonotic origin are generally triggered by disturbances in the dynamics of interactions between populations of humans, infectious agents, animal reservoirs, and sometimes insect vectors. By varying their habitat or abundance, environmental and socioeconomic changes can modify the probabilities of interactions between these populations.

The link between human intrusion into an ecosystem and the onset of an epidemic is illustrated well by the case of the human immunodeficiency virus. Its emergence was probably due to an increase in hunting and consumption of chimpanzee meat in the region of Kinshasa (Democratic Republic of the Congo) between 1920 and 1950: increased contacts between humans and primates infected by simian immunodeficiency viruses promoted adaptation of this pathogen to humans (106). The impact of declining biodiversity due to fragmentation of habitats and of urbanization on the frequency of infections has also been well documented, particularly for Lyme disease (107). Agricultural activities are associated with 25% of all emerging infectious agents and almost 50% of emerging zoonoses (108). The role of farms and live animal markets as incubators for the virus has been demonstrated in the case of influenza (109). Hence the growing interest in eco-epidemiology, a new discipline that cuts across ecology, epidemiology and biomedical sciences.

The Manhattan Principles also underscore the need for holistic and prospective approaches to emerging infectious diseases that take the complex interconnections between species into account. Good management of an epidemic in any given country is built on the socioeconomic, political, religious and cultural realities that prevail there. And the support of populations for public health strategies is also essential. Strategy, communication and education strategies must adapt to each societal context. The conclusion delivered in the Manhattan congress summary is unequivocal: “*We must design adaptive, forward-looking and multidisciplinary solutions to the challenges that undoubtedly await us*.”

The concept of “One Health” based on the 12 Manhattan Principles was introduced in 2008 in Sharm el-Sheik (Egypt), during a symposium on infectious risks linked to contact between human and animal ecosystems (105). Today, it dominates the communications of international public health organizations such as the World Organization for Animal Health, the Food and Agriculture Organization, the WHO and the US Centers for Disease Control and Prevention. Other similar concepts have emerged (110), such as EcoHealth and Planetary Health. EcoHealth emphasizes the importance of conserving ecosystems in order to protect animal and human health. Planetary Health adopts a more global vision and includes reflection on a more sustainable economy due to the interconnectedness of global threats. For example, it is now well established that climate change affects the distribution of a wide range of vector-borne diseases, and will continue to do so in the decades to come (111).

The One Health concept and its enshrinement in the EcoHealth and Planetary Health approaches takes into account the interconnections between living things, but also the necessary abandonment of linear and reductive thinking, and has adopted a decompartmentalization of disciplines. It will undoubtedly help us better understand, prevent and respond to global health crises in the future.

### Is Public Health Based on the One Health Principle Compatible with Liberalism?

Though the One Health concept has come to dominate the communications of national and international public health organizations, it appears to be only rarely integrated into liberal democratic government decision-making. There are many possible reasons for this and we only highlight the most important ones.

As mentioned previously, political decision-makers, who are mostly trained in the human sciences, sorely lack scientific knowledge and tend to adopt a constructivist/relativist view of knowledge. But another major problem is the fact that a One Health approach involves the prevention of threats by acting on the socioeconomic conditions conducive to their emergence, and this implies much greater regulation of the economy by states, which is the exact opposite of what liberal economists recommend. For example, the Austrian economist Friedrich Hayek states that the role of the state must be limited to providing an “*adequate organization of certain institutions like money, markets and channels of communication*,” and to creating “*a legal system designed both to preserve competition and make it operate as beneficially as possible*” (112). In addition, by considering the free market as the only engine of the economy, liberals tend to privilege the production of an exchange value, money, and to neglect all processes which are not directly market-related and monetizable, starting with the environment, health and even human life.

Historically, liberal economics pioneers have progressively neglected the role of natural resources in the wealth of a nation. The French economist François Quesnay, and more broadly the physiocratic economic school of thought, emphasized the role of land as the main production factor. Later, the English economists Adam Smith and David Ricardo named the three production factors needed in order to create wealth: land (by extension, natural resources), capital and labor. Yet, quickly, classical economists stopped being interested in nature and natural resources. In 1803, the French economist Jean-Batiste Say wrote “*natural resources are infinite because, if they weren't, we would not obtain them freely. Since they cannot be multiplied, nor exhausted, they cannot be the object of economic sciences*” (113). Thus, modern economic liberalism often places little value on the environment, except for exploitable resources which are clearly limited and can be converted into currency.

The privatization of health services in order to submit them to the free market was initiated in the early 1980s by Ronald Reagan in the United States (114) and Margaret Thatcher in the United Kingdom (115) and then continued in many European states. This privatization policy cannot be considered as a “specific political choice”, which would only reflect the non-ideological preferences of certain political leaders. For example, Calum Paton (116) show that the market reform of the English National Health Service “*has derived from the ideological hegemony of a naive anti-statism (hostility to a misleadingly defined and often mythological ‘centralist state') in public services and enthusiasm for market competition*”. This liberal *laisser-faire* policy had some consequences for public health. It has helped gradually disconnect economic growth from improved public health by reducing access to healthcare to those who can pay which had dramatic consequences. For example, life expectancy in the United States, where health systems are private, is lower than that of many countries with lower gross domestic product per capita but whose health services have not been privatized (83, 117). This resulted in the establishment of just-in-time management, which severely reduced the capacity of health services to cope with a massive influx of patients during the Covid-19 pandemic (118). In Italy, for example, years of austerity, regionalization and privatization of healthcare systems caused them to collapse in the face of Covid-19 and led to a sharp rise in the death toll due to insufficient access to healthcare (119). The Italian situation is in striking contrast to the strong public health systems of Hong Kong, Singapore and Japan that have shown great resilience and efficiency in managing the Covid-19 outbreak (120). This leads several authors (121, 122) to conclude that privatization of health services and individualization of risks might undermine our ability to address future pandemics and to call for the refinancing of public health services.

In the face of a global threat, when human life is endangered and protection of health or the environment becomes a priority, the logic of the free market which defines the priorities of liberal governance is inadequate. Indeed, neither the environment nor human life can be reduced to a monetary value and therefore they cannot be considered as legitimate priorities for liberalism. Thus, lockdowns to protect lives have been viewed with great reluctance by liberal governments due to their highly negative impacts on markets and the economy. They have even been violently rejected by many liberal political leaders. Faced with the progression of the pandemic, US President Trump tweeted on March 23, 2020 that “*We cannot let the cure be worse than the problem itself* ” and Brazilian President Bolsonaro declared on March 25, 2020 that “*Our lives have to go on. Jobs must be kept. We must, yes, get back to normal*.” This refusal to make public health a priority is therefore not the consequence of a failure of leadership but of a structural inability of liberalism to value anything other than the economy. This lack of neutrality toward the economy stems from the liberal belief that trade is supposed to be the only thing that connects and unites the members of a society that liberalism sees as competitors with potentially conflicting interests. It is therefore not surprising to see, in the midst of the Covid-19 crisis, liberal economists proposing theories to justify a lack of action to protect health. For example, Miles et al. conclude that, by monetizing human life, containment strategies come at far too high an economic cost (123).

Finally, the greatest obstacle to the application of the One Health principle is the absence of an international institution with both scientific expertise and an intervention capacity and this situation is the consequence of an international order inspired by liberal ideology. The WHO was founded in 1948 following the Spanish flu pandemic which demonstrated the need for international pandemic management. However, the WHO, as a United Nations (UN) branch, is part of the international liberal order rooted in the “Peace of Westphalia” (124). This treaty, who end up the Thirty Years' War, is the corner stone of the modern international relations. The principles, which are clearly laid out by Henry Kissinger in the seminal book World Order (125), put forward the principle of equality between sovereign states “*regardless of their power or domestic system, was instituted*”. Related to this, the “Peace of Westphalia” contribute to implement a liberal approach about religion and everything that may concern a definition of the common good. As Kissinger pointed out: “*The concept of state sovereignty was established. The right of each signatory to choose its own domestic structure and religious orientation free from intervention was affirmed, while novel clauses ensured that minority sects could practice their faith in peace and be free from the prospect of forced conversion. Beyond the immediate demands of the moment, the principles of a system of ‘international relations' were taking shape, motivated by the common desire to avoid a recurrence of total war on the Continent*”. “*If a state would accept these basic requirements, it could be recognized as an international citizen able to maintain its own culture, politics, religion, and internal policies, shielded by the international system from outside intervention*”. The governance of the international order is based on organizations, forum, etc., in which sovereign states try to deliberate, to reach consensus and expose their dissension. Consequently, WHO have no possibility to impose any kind of “rules” including rules derived from One Health principle. Thus, the concrete application of the One Health principle at global level implies nothing less than a revolution in the liberal-inspired international order.

### Limitations of Our Study

The current research has several obvious limitations. First, as the vast majority of liberal democratic governments have failed, with varying degrees, to effectively manage the COVID-19 pandemic, we have chosen a general approach to analyze the reasons for this failure. This choice makes us run the risk of caricaturing the management that has been done by certain countries. Secondly, it is often difficult to know the real motivations of a political decision. These are often multiple and therefore complex. Therefore, a detailed retrospective analysis, country by country, would be essential to validate our hypothesis but this is beyond our scope. It is generally accepted that ideologies act as a filter of perception. Our analysis simply suggests that the liberal filter has to be explore in order to explain some political choices. Third, there is no agreed definition of liberalism or postmodernism. Accordingly, we have introduced our own definitions of these terms to make it clear what we are talking about. However, these definitions may not be accepted and lead to the rejection of our conclusions. Fourth, our hypothesis cannot entirely satisfy the Popperian criterion for refutation. The issue we are analyzing is both too complex to be modeled and no experimental approach is possible. Our hypothesis can therefore only be evaluated by its coherence and its explanatory capacity. This situation, however, is not uncommon in science.

## Conclusions

The SARS-CoV-2 pandemic represents the first major health crisis of the 21st century, and is considered the most serious pandemic since the Spanish flu of 1918. Its human and economic toll will probably be considerable and will have profound repercussions in the decades to come. In the United States alone, the total cost of the Covid-19 pandemic is estimated at more than $16 trillion (21). Although the infectious agent was quickly identified and diagnostic tests were readily available (1), Western governments have, in the opinion of many scientists (11–13, 126), made many tragic management errors in their handling of the pandemic. Retrospectively, only a very small number of countries in the world were able to respond effectively. These countries were not the richest, did not have better medical expertise, and were not countries with authoritarian governments; they just made the right decisions. Blaming the failures of all Western governments on the incompetence of their leaders alone makes little sense. Several studies (100, 101) have analyzed the impact of culture, and have highlighted the advantages of collectivist cultures over individualistic Western cultures. We argue that we must also question the dominant ideological framework supporting political action of Western governments.

**Figure 4:**
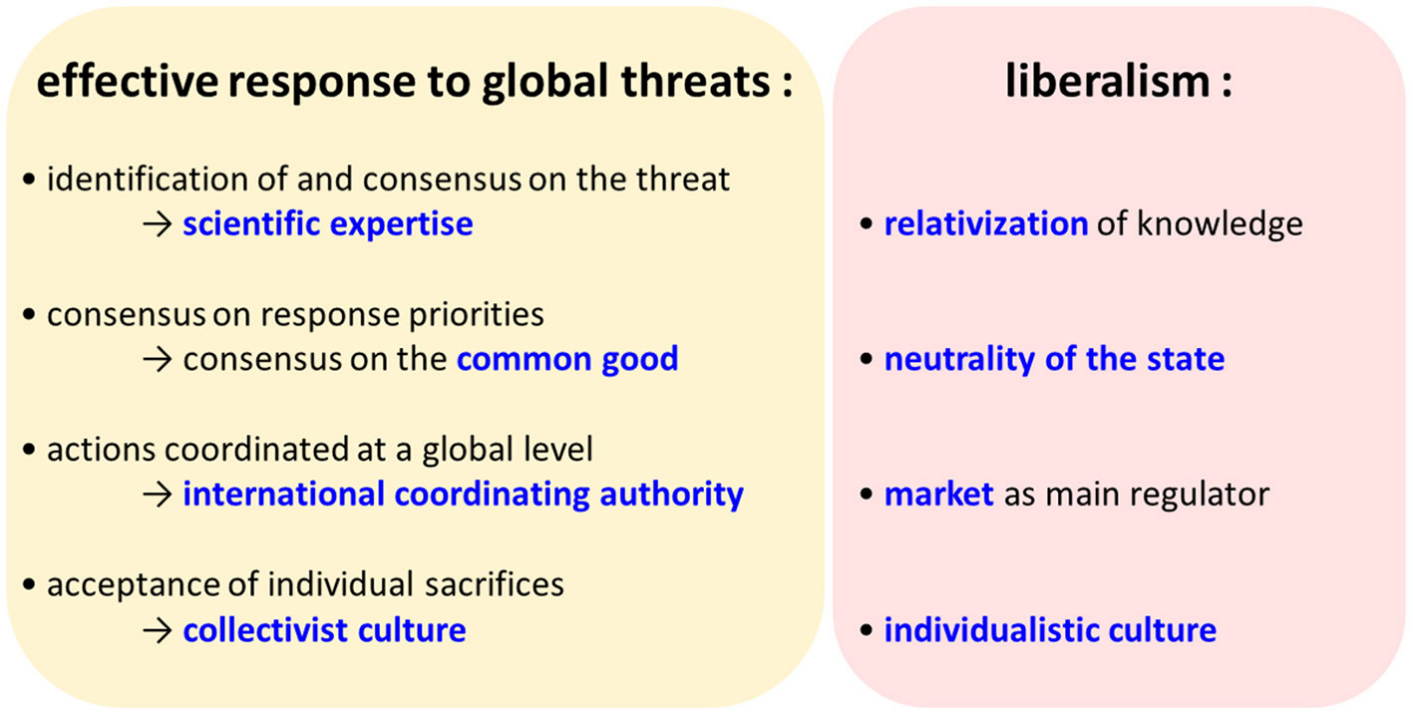
Structural weaknesses of liberalism in the face of global threats.

Liberalism dominates Western societies and is often considered as devoid of axioms or based on established truths, which leads to the view that governance failures are necessarily attributable to political decision-makers. However, as discussed above, liberalism is not devoid of axioms. Its conception of the neutrality of the state and faith in competition as a self-organizing principle tends to favor a relativism of knowledge, and this is not conducive to policies led by science, which has been identified as essential for the management of global threats. Liberalism also affirms the predominance of the individual over society as a whole and thus hampers the effective implementation of policies involving a collective effort and a sacrifice of individual interests in favor of the common good. Finally, liberalism considers that the economy, and not public health or preservation of the environment, constitutes the top priority. This prioritization makes little sense in the face of the obvious interconnectedness of ecosystems, animal and human health and the economy. Consequently, our analysis suggests that liberal ideology is particularly ill-suited to the management of global threats (Figure 4). This is particularly worrying given that Western countries see themselves as the main leaders in the fight against climate change and pollution. Thus, it is urgent that we develop a new conceptual framework for governance that is more in line with our scientific knowledge and that allows us to rapidly address global threats as they emerge.

If we view the COVID-19 crisis as a crash test, we can only conclude that our capacity to react to global threats is very weak. Without a vaccine or specific treatment, reducing social contacts is our only possible measure to limit the spread of emerging pathogens even though it is tragically insufficient and economically very costly. The development and validation of vaccines against COVID-19 in only 1 year (1), compared with the several years or even decades which are generally required (127), was a true organizational feat. However, it will clearly take much more than two years to fully vaccinate the entire planet. In the span of 1 year, only 56% of the world's population had received at least one dose of the vaccine (128). In addition, a decline in the immunity conferred by vaccines over time (129) and the emergence of vaccine-escaping variants (130) could complicate the process. The weakness of our capacity to respond to the consequences of climate change, the decline in biodiversity and atmospheric and plastic pollution will be even greater. Indeed, there will be no technological miracle solution equivalent to a vaccine to cope with these global threats, whose consequences may be irreversible and affect future generations. Consequently, we must not only react to these threats. We must prevent them by acting on the conditions of their emergence, by adapting our socioeconomic systems and preserving ecosystems around the world. Preventing threats would be less expensive (131) and especially less risky than “*laissez-faire”*. For example, it has been estimated that the costs of preventing the risks of a pandemic for 10 years would only amount to about 2% of the world costs of the COVID-19 pandemic in 2020 (132). This would also probably be a way of addressing the demands of economists, such as Joseph Stiglitz, to combine free competition, which generates societal dynamism and innovation, with regulation in favor of the common good.

Time is running out. The COVID-19 crisis has clearly shown that we are not all equal when it comes to the consequences of global threats (133). Future climate change is predicted to increase inequalities (134, 135) and the risk of violent conflict (136) within societies and between countries. Ultimately, this could jeopardize all attempts at international cooperation, which is so essential to fighting global threats. Moreover, when it comes to climate change, experts agree that we are dangerously close to the tipping point (137). Going beyond it would lead to irreversible cascading changes, the consequences of which could be appalling.

Considering the dramatic and unprecedented challenges facing mankind today, it is necessary to rethink liberalism and its scope. Giving greater importance to science in the political decision-making process and establishing a *modus vivendi* based on One Health-like approaches could be a promising avenue for reflection on how to deal with phenomena which jeopardize the very survival of our species. This could constitute a basis for building a form of governance suitable for the globalized world of the 21^st^ century.

In order to avoid any misunderstanding, we wish to emphasize that this does not constitute an advocacy for the abandonment of liberalism nor a promotion of authoritarianism. We have clearly underlined, by examining the examples of Turkey and Iran, the poor results of authoritarian governments. Studies on collectivist cultures show that the effectiveness of a health policy depends on the support of the greatest number. Moreover, we suggest to privilege a modus vivendi inspired by a key liberal author – Thomas Hobbes. Finally, our critique of liberalism is limited to its handling of global threats. Our conclusion is therefore not in favor of an abandonment of liberalism but rather of a return to its historical roots.

On the whole, our analysis suggests that because liberal ideology provides its own definitions of the common good and the place of scientific knowledge in the governance process and can thus affect the response to global threats, it should be urgently taken into consideration by public health experts.

## Author Contributions

EM and PN wrote the first draft of the manuscript. JP contributed some sections of the manuscript. All authors read and approved the submitted version.

## Conflict of Interest

The authors declare that the research was conducted in the absence of any commercial or financial relationships that could be construed as a potential conflict of interest.

## Publisher's Note

All claims expressed in this article are solely those of the authors and do not necessarily represent those of their affiliated organizations, or those of the publisher, the editors and the reviewers. Any product that may be evaluated in this article, or claim that may be made by its manufacturer, is not guaranteed or endorsed by the publisher.

## References

[1] Carvalho T Krammer F Iwasaki A. The first 12 months of COVID-19: a timeline of immunological insights. Nat Rev Immunol. (2021) 21:245–56. 10.1038/s41577-021-00522-1 33723416 PMC7958099

[2] Wang H Paulson KR Pease SA Watson S Comfort H Zheng P. Estimating excess mortality due to the COVID-19 pandemic: a systematic analysis of COVID-19-related mortality, 2020–21. Lancet. (2022) 399:1513–36. 10.1016/s0140-6736(21)02796-3 35279232 PMC8912932

[3] Liu J Zhang L Yan Y Zhou Y Yin P Qi J. Excess mortality in Wuhan city and other parts of China during the three months of the covid-19 outbreak: findings from nationwide mortality registries. BMJ. (2021) 372. 10.1136/bmj.n415 33627311 PMC7900645

[4] Michaela L Marriet M Smits V Koolman X. The Dutch COVID-19 approach : Regional differences in a small country. Heal Policy Technol. (2020) 9:613–22. 10.1016/j.hlpt.2020.08.008 32874861 PMC7450952

[5] Horton R. Offline: COVID-19—a reckoning. Lancet. (2020) 395:935. 10.1016/S0140-6736(20)30669-3 32199478 PMC7156226

[6] Haffajee RL. Mello MM. Thinking globally, acting locally - the US response to COVID-19. N Engl J Med. (2020) 382:e75. 10.1056/NEJMp2006740 32240580

[7] Berlivet L Löwy I. Hydroxychloroquine controversies: clinical trials, epistemology, and the democratization of science. Med Anthropol Q. (2020) 34:525–41. 10.1111/maq.12622 33210338 PMC7753536

[8] Tanne JH. Covid-19: Trump is criticised for again promoting unorthodox medical information. BMJ. (2020) 370:m3046. 10.1136/bmj.m3046 32732320

[9] Idrovo AJ Manrique-Hernández EF Fernández Niño JA. Report from bolsonaro's brazil: the consequences of ignoring science. Int J Heal Serv. (2021) 51:31–6. 10.1177/0020731420968446 33100167 PMC7756057

[10] Taylor L. “We are being ignored”: Brazil's researchers blame anti-science government for devastating COVID surge. Nature. (2021) 593:15–6. 10.1038/d41586-021-01031-w 33907333

[11] Horton R. The COVID-19 Catastrophe: What's Gone Wrong and How to Stop it Happening Again. Polity Pre Cambridge, UK and Medford, MA: John Wiley & Sons. (2020).

[12] Now S. Scientific american endorses joe biden. Sci Am. (2020) 323:12–3. 10.1038/scientificamerican1020-12

[13] Editors T. Dying in a leadership vacuum. N Engl J Med. (2020) 383:1479–80. 10.1056/NEJMe2029812 33027574

[14] Kuznetsova L. COVID-19: the world community expects the world health organization to play a stronger leadership and coordination role in pandemics control. Front Public Heal. (2020) 8:1–6. 10.3389/fpubh.2020.00470 33014970 PMC7505920

[15] Atwoli L Baqui AH Benfield T Bosurgi R Godlee F Hancocks S. Call for emergency action to limit global temperature increases, restore biodiversity and protect health. Lancet Reg Heal Eur. (2021) 9:100220. 10.1016/j.lanepe.2021.100220 34514455 PMC8418936

[16] Snyder-beattie AE Ord T Bonsall MB. An upper bound for the background rate of human extinction. Sci Rep. (2019) 9:11054. 10.1038/s41598-019-47540-7 31363134 PMC6667434

[17] Brockmann D Helbing D. The hidden geometry of complex, network-driven contagion phenomena. Science. (2013) 342:1337–42. 10.1126/science.1245200 24337289

[18] Poutanen SM Low DE Henry B Finkelstein S Rose D Green K. Identification of severe acute respiratory syndrome in Canada. N Engl J Med. (2003) 348:1995–2005. 10.1056/NEJMoa030634 12671061

[19] Girard MP Tam JS Assossou OM Kieny MP. The 2009 A (H1N1) influenza virus pandemic: a review. Vaccine. (2010) 28:4895–902. 10.1016/j.vaccine.2010.05.031 20553769

[20] Lee J-W McKibbin WJ. Globalization and disease: The case of SARS. Asian Econ Pap. (2004) 3:113–31. 10.1162/1535351041747932

[21] Cutler DM Summers LH. The COVID-19 pandemic and the $16 trillion virus. JAMA. (2020) 324:333–60. 10.1001/jama.2020.19759 33044484 PMC7604733

[22] There's money in the air: the CFC ban and Dupont's regulatory strategy. Bus Strateg Environ. (1997) 6:276–286. 10.1002/(SICI)1099-0836(199711)6:5&lt;276::AID-BSE123&gt;3.0.CO;2-A

[23] Milina M Rowland F. Stratospheric sink for chlorofluoromethanes: chlorine atom-catalysed destruction of ozone. Nature. (1974) 249:810–2. 10.1038/249810a0

[24] Farman J Gardiner B Shanklin J. Large losses of total ozone in Antarctica reveal seasonal ClOx/NOx interaction. Nature. (1985) 315:207–10. 10.1038/315207a0

[25] baron de Montesquieu CD. De l'esprit des lois. Geneva: Barrillot & Fils (1872).

[26] Helvétius C-A. De l'esprit. Paris: Durand (1758).

[27] Smith A. An Inquiry into the Nature and Causes of the Wealth of Nations., eds. Strahan T. Cadell. London: Methuen & Co (1776). 10.1093/oseo/instance.00043218

[28] Rawls J. Theory of Justice. Cambridge: Harvard University Press (1973).

[29] Larmore C. Political liberalism. Polit Theory. (1990) 18:339–60. 10.1177/0090591790018003001

[30] Larmore C. Patterns of Moral Complexity. Cambridge: Cambridge University Press (1987). 10.1017/CBO9780511625107

[31] Kymlicka W. Liberal individualism and liberal neutrality. Ethics. (1989) 99:883–905. 10.1086/29312511882210

[32] Schumpeter J. Capitalism, Socialism and Democracy. New York, NY: Harper & Brothers (1942).

[33] Moore M. Pluralism, relativism, and liberalism. Polit Res Q. (2009) 62:244–56. 10.1177/1065912908320666

[34] Kahan DM Jenkins-Smith H Braman D. Cultural cognition of scientific consensus. J Risk Res. (2011) 14:147–74. 10.1080/13669877.2010.511246

[35] Diethelm PA Rielle JC McKee M. The whole truth and nothing but the truth? The research that philip morris did not want you to see. Lancet. (2005) 366:86–92. 10.1016/S0140-6736(05)66474-4 15993237

[36] Falkner R. Business Power And Conflict In International Environmental Politics. Macmillan Basingstoke, UK: Palgrave. (2008). 10.1057/9780230277892

[37] Caney S. Consequentialist defences of liberal neutrality. Philos Q. (1991) 41:457–77. 10.2307/2220080

[38] Sur RL Dahm P. History of evidence-based medicine. Indian J Urol. (2011) 27:487–9. 10.4103/0970-1591.91438 22279315 PMC3263217

[39] Sackett D Straus S Richardson W. Evidence-based Medicine: How to Practice and Teach EBM. Edinburgh: Churchill Livingstone. (2000).

[40] Berlin JA Golub RM. Meta-analysis as evidence: building a better pyramid. JAMA. (2014) 312:603–5. 10.1001/jama.2014.8167 25117128

[41] Sciama Y. Is France's president fueling the hype over an unproven coronavirus treatment? Science. (2020) 10. 10.1126/science.abc1786

[42] Mary C. Sound and fury in the microbiology lab. Science. (2012) 336:155. 10.1126/science.336.6078.155-a 22383821

[43] Mucchielli L. Behind the French controversy over the medical treatment of COVID-19: The role of the drug industry. J Sociol. (2020) 56:736–44. 10.1177/1440783320936740

[44] Stengers I. Que nous apprend la pandémie?. Esprit. (2021) Mars:37–47. 10.3917/espri.2015.0037

[45] Popper KR. The Logic Of Scientific Discovery. London: Hutchinson & Co. (1959). 10.1063/1.3060577

[46] Merton RK. The Normative Structure of Science. Chicago, IL: University of Chicago Press (1942). Available online at: http://eprints.lse.ac.uk/42339/ (accessed June 14, 2022).

[47] Lyotard J-F. La Condition Postmoderne. Paris: Les éditions de minuit. (1979).

[48] Kuhn TS. The Structure of Scientific Revolutions. Chicago, IL: University of Chicago Press (1962).

[49] Foucault M. Les mots et les choses: Une archéologie des sciences humaines. Paris: Éditions Gallimard (1966).

[50] Harding S. The Science Question in Feminism. New York, NY: Cornell University Press (1986).

[51] Rorty R. Contingency, Irony, and Solidarity. Cambridge: Cambridge University Press (1989). 10.1017/CBO9780511804397

[52] Gibbons M Limoges C Nowotny H Schwartzman S Scott P Trow M. The New Production of Knowledge: The Dynamics of Science and Research in Contemporary Societies. London: Sage (1994).

[53] Etzkowitz H Leydesdorff L. The dynamics of innovation : from national systems and “‘Mode 2”' to a triple helix of university – industry – government relations. Res Policy. (2000) 29:109–23. 10.1016/S0048-7333(99)00055-4

[54] Carayannis EG Campbell DFJ. Mode 3 knowledge production in quadruple helix innovation systems. In: Mode 3 Knowledge Production in Quadruple Helix Innovation Systems. New York, NY: Springer. (2012)

[55] Muraille E. Ethical control of innovation in a globalized and liberal world: is good science still science? Endeavour. (2019) 43:100709. 10.1016/j.endeavour.2020.100709 32115245

[56] Friedman M. Capitalism and Freedom. Chicago, IL: University of Chicago Press (1962).

[57] McGovern P Yacobucci P. A Postmodern present and president: postmodernity, political science and the trump presidency. J Polit Sci Educ. (2021) 17:938–54. 10.1080/15512169.2021.1921587

[58] Polletta F Callahan J. Deep stories, nostalgia narratives, and fake news: storytelling in the trump era. Am J Cult Sociol. (2017) 5:392–408. 10.1057/s41290-017-0037-7

[59] Kien G. Postmodernism trumps all: the world without facts. Qual Inq. (2021) 27:374–80. 10.1177/1077800420918892

[60] Druckman JN Klar S Krupnikov Y Levendusky M Ryan JB. Affective polarization, local contexts and public opinion in America. Nat Hum Behav. (2021) 5:28–38. 10.1038/s41562-020-01012-5 33230283

[61] Kahane LH. Politicizing the mask: political, economic and demographic factors affecting mask wearing behavior in the USA. East Econ J. (2021) 47:163–83. 10.1057/s41302-020-00186-0 33424048 PMC7783295

[62] Flores A Cole JC Dickert S Eom K Jiga-Boy GM Kogut T. Politicians polarize and experts depolarize public support for COVID-19 management policies across countries. Proc Natl Acad Sci U S A. (2022) 119:e2117543119. 10.1073/pnas.2117543119 35042779 PMC8784107

[63] Szahaj A. Postmodern liberalism as a new humanism. Diogenes. (2005) 206:63–70. 10.1177/0392192105052622

[64] Laclau E Mouffe C. Hegemony and Socialist Strategy. Towards a radical Democratic Politics London: Verso. (2014).

[65] Snow CP. The Two Cultures and the scientific revolution. London: Cambridge University Press. (1959).

[66] Caplan N. The two-communities theory and knowledge utilization. Am Behav Sci. (1979) 22:459–70. 10.1177/000276427902200308

[67] Weiss CH. The Many Meanings of Research Utilization. Public Adm Rev. (1979) 39, 426–31. 10.2307/3109916

[68] Miller JD Scott EC Okamoto S. Public acceptance of evolution. Science. (2006) 313:765–6. 10.1126/science.1126746 16902112

[69] Hameed S Hameed S Oktar A Oktar A. Bracing for islamic creationism. Science. (2008) 322:1637–8. 10.1126/science.1163672 19074331

[70] Ong E Glantz S. Tobacco industry effort subverting the international agency for research on cancer's second-hand smoke study. Lancet. (2000) 355:1253–9. 10.1016/S0140-6736(00)02098-5 10770318

[71] Bekelman JE Li Y Gross CP. Scope and impact of financial conflicts of interest in biomedical research. JAMA. (2003) 289:454–65. 10.1001/jama.289.4.454 12533125

[72] Bailey CS Fehlings MG Rampersaud YR Hall H Wai EK Fisher CG. Industry and evidence-based medicine: believable or conflicted? A systematic review of the surgical literature. Can J Surg. (2011) 54:321–6. 10.1503/cjs.008610 21933525 PMC3195655

[73] Séralini GE Mesnage R Defarge N Spiroux de Vendômois J. Conflicts of interests, confidentiality and censorship in health risk assessment: the example of an herbicide and a GMO. Environ Sci Eur. (2014) 26:1–6. 10.1186/s12302-014-0013-6 27752411 PMC5044951

[74] Reed G Hendlin Y Desikan A MacKinney T Berman E Goldman GT. The disinformation playbook: how industry manipulates the science-policy process—and how to restore scientific integrity. J Public Health Policy. (2021) 42:622–34. 10.1057/s41271-021-00318-6 34811464 PMC8651604

[75] Goldberg RF Vandenberg LN. The science of spin: targeted strategies to manufacture doubt with detrimental effects on environmental and public health. Environ Heal A Glob Access Sci Source. (2021) 20:1–11. 10.1186/s12940-021-00723-0 33771171 PMC7996119

[76] Hotez PJ. Anti-science extremism in America: escalating and globalizing. Microbes Infect. (2020) 22:505–7. 10.1016/j.micinf.2020.09.005 32961275

[77] Nogrady B. Scientists under attack. Nature. (2021) 598:250–253. 10.1038/d41586-021-02741-x 34645996

[78] Hotez PJ. Mounting antiscience aggression in the United States. PLoS Biol. (2021) 19:2–5. 10.1371/journal.pbio.3001369 34319972 PMC8351985

[79] The challenge of the post-truth era. Nat Cell Biol. (2018) 20:1231. 10.1038/s41556-018-0231-z 30361700

[80] Cohen J. Calm down about political ‘mischief' around COVID-19 vaccines, scientists say. Science. (2020) 10.1126/science.abf1577

[81] Editorial. Trumping science. Nat Biotechnol. (2020) 38:1105. 10.1038/s41587-020-0713-y 32973360 PMC7513682

[82] Piller C. Undermining CDC. Science. (2020) 370:394–9. 10.1126/science.370.6515.394 33093092

[83] Woolhandler S Himmelstein DU Ahmed S Bailey Z Bassett MT Bird M. Public policy and health in the Trump era. Lancet. (2021) 397:705–53. 10.1016/S0140-6736(20)32545-9 33581802

[84] Brigitte et Emmanuel Macron de retour au théâtre pour ≪ une séance de psy jubilatoire ≫,. Gala. (2020). Available online at: https://www.gala.fr/l_actu/news_de_stars/brigitte-et-emmanuel-macron-de-retour-au-theatre-pour-une-seance-de-psy-jubilatoire_444423 (accessed June 14, 2022).

[85] Thorp HH. Trump lied about science. Science. (2020) 369:1409. 10.1126/science.abe7391 32917826

[86] Soper GA. The lessons of the pandemic. Science. (1919) 49:501–6. 10.1126/science.49.1274.501 17793800

[87] Hellewell J Abbott S Gimma A Bosse NI Jarvis CI Russell TW. Feasibility of controlling COVID-19 outbreaks by isolation of cases and contacts. Lancet Glob Heal. (2020) 8:e488–96. 10.1016/S2214-109X(20)30074-7 32119825 PMC7097845

[88] Gostic KM Gomez ACR Mummah RO Kucharski AJ Lloyd-Smith JO. Estimated effectiveness of symptom and risk screening to prevent the spread of COVID-19. Elife. (2020) 9:1–18. 10.7554/eLife.55570 32091395 PMC7060038

[89] Abdullah ASM Tomlinson B Cockram CS Thomas GN. Lessons from the severe acute respiratory syndrome outbreak in Hong Kong. Emerg Infect Dis. (2003) 9:1042–5. 10.3201/eid0909.030366 14519237 PMC3016765

[90] Fraser C Riley S Anderson RM Ferguson NM. Factors that make an infectious disease outbreak controllable. Proc Natl Acad Sci U S A. (2004) 101:6146–51. 10.1073/pnas.0307506101 15071187 PMC395937

[91] Peak CM Childs LM Grad YH Buckee CO. Comparing nonpharmaceutical interventions for containing emerging epidemics. Proc Natl Acad Sci U S A. (2017) 114:4023–8. 10.1073/pnas.1616438114 28351976 PMC5393248

[92] Mazey S Richardson J. Lesson-drawing from New Zealand and COVID-19: the need for anticipatory policy making. Polit Q. (2020) 91:561–70. 10.1111/1467-923X.12893 32836413 PMC7436465

[93] Wilson S. Pandemic leadership: Lessons from New Zealand's approach to COVID-19. Leadership. (2020) 16:279–93. 10.1177/174271502092915134562101

[94] Dighe A Cattarino L Cuomo-Dannenburg G Skarp J Imai N Bhatia S. Response to COVID-19 in South Korea and implications for lifting stringent interventions. BMC Med. (2020) 18:1–12. 10.1186/s12916-020-01791-8 33032601 PMC7544529

[95] Summers DJ Cheng DHY Lin PHH Barnard DLT Kvalsvig DA Wilson PN. Potential lessons from the Taiwan and New Zealand health responses to the COVID-19 pandemic. Lancet Reg Heal - West Pacific. (2020) 4:100044. 10.1016/j.lanwpc.2020.100044 34013216 PMC7577184

[96] San S Bastug MF Basli H. Crisis management in authoritarian regimes: A comparative study of COVID-19 responses in Turkey and Iran. Glob Public Health. (2021) 16:485–501. 10.1080/17441692.2020.1867880 33378230

[97] Liu Y Morgenstern C Kelly J Lowe R Munday J Villabona-Arenas CJ. The impact of non-pharmaceutical interventions on SARS-CoV-2 transmission across 130 countries and territories. BMC Med. (2021) 19:1–12. 10.1186/s12916-020-01872-8 33541353 PMC7861967

[98] Brauner JM Mindermann S Sharma M Johnston D Salvatier J Gavenčiak T. Inferring the effectiveness of government interventions against COVID-19. Science. (2021) 371:eabd9338. 10.1126/science.abd9338 33323424 PMC7877495

[99] Bavel JJV Baicker K Boggio PS Capraro V Cichocka A Cikara M. Using social and behavioural science to support COVID-19 pandemic response. Nat Hum Behav. (2020) 4:460–71. 10.1038/s41562-020-0884-z 32355299

[100] Maaravi Y Levy A Gur T Confino D Segal S. “The tragedy of the commons”: how individualism and collectivism affected the spread of the COVID-19 pandemic. Front Public Heal. (2021) 9:1–6. 10.3389/fpubh.2021.627559 33643992 PMC7905028

[101] Liu JH. Majority world successes and European and American failure to contain COVID-19 : cultural collectivism and global leadership. Asian J Soc Psychol. (2021) 24:23–9. 10.1111/ajsp.12461 33821138 PMC8014605

[102] Whitmee S Haines A Beyrer C Boltz F Capon AG De Souza Dias BF. Safeguarding human health in the anthropocene epoch: report of the rockefeller foundation-lancet commission on planetary health. Lancet. (2015) 386:1973–2028. 10.1016/S0140-6736(15)60901-1 26188744

[103] Neal P. Vulgar liberalism. Polit Theory. (1993) 21:623–42. 10.1177/0090591793021004004

[104] Ruwen O. La Panique morale. Paris: Grasset. (2004).

[105] Zinsstag J Schelling E Waltner-Toews D Tanner M. From “one medicine” to “one health” and systemic approaches to health and well-being. Prev Vet Med. (2011) 101:148–56. 10.1016/j.prevetmed.2010.07.003 20832879 PMC3145159

[106] Faria NR Rambaut A Suchard MA Baele G Bedford T Ward MJ. The early spread and epidemic ignition of HIV-1 in human populations. Science. (2014) 346:56–61. 10.1126/science.1256739 25278604 PMC4254776

[107] Keesing F Belden LK Daszak P Dobson A Harvell CD Holt RD. Impacts of biodiversity on the emergence and transmission of infectious diseases. Nature. (2010) 468:647–52. 10.1038/nature09575 21124449 PMC7094913

[108] Rohr JR Barrett CB Civitello DJ Craft ME Delius B DeLeo GA. Emerging human infectious diseases and the links to global food production. Nat Sustain. (2019) 2:445–56. 10.1038/s41893-019-0293-3 32219187 PMC7091874

[109] Sims L Peiris M. One health: the hong kong experience with avian influenza. In: One Health: The Human-Animal-Environment Interfaces in Emerging Infectious Diseases. Berlin: Springer. (2012). p. 281–298. 10.1007/82_2012_254

[110] Lerner H Berg C. A comparison of three holistic approaches to health: one health, ecohealth, and planetary health. Front Vet Sci. (2017) 4:1–7. 10.3389/fvets.2017.00163 29085825 PMC5649127

[111] Semenza JC Suk JE. Vector-borne diseases and climate change: a European perspective. FEMS Microbiol Lett. (2018) 365:1–9. 10.1093/femsle/fnx244 29149298 PMC5812531

[112] Hayek F. The Road to Serfdom. London: Routledge Press (1944).

[113] Say J-B. Cours Complet d'economie Politique Pratique. Tome II: Economica. (2009).

[114] Larsen LT Stone D. Governing health care through free choice: neoliberal reforms in Denmark and the United States. J Health Polit Policy Law. (2015) 40:941–70. 10.1215/03616878-3161162 26195602

[115] Klein R. The twenty-year war over England's national health service: A report from the battlefield. J Health Polit Policy Law. (2013) 38:849–69. 10.1215/03616878-2210503 23645869

[116] Paton C. Garbage-can policy-making meets neo-liberal ideology: twenty five years of redundant reform of the english national health service. Soc Policy Adm. (2014) 48:319–42. 10.1111/spol.12044

[117] Jackson T. Prosperity Without Growth: Foundations for the Economy of Tomorrow. Routledge. (2017).

[118] Navarro V. The consequences of neoliberalism in the current pandemic. Int J Heal Serv. (2020) 50:271–5. 10.1177/0020731420925449 32380877 PMC7218352

[119] Buzelli ML Boyce T. The privatization of the italian national health system and its impact on health emergency preparedness and response: the COVID-19 case. Int J Heal Serv. (2021) 51:501–8. 10.1177/00207314211024900 34125625

[120] Legido-Quigley H Asgari N Teo YY Leung GM Oshitani H Fukuda K. Are high-performing health systems resilient against the COVID-19 epidemic? Lancet. (2020) 395:848–50. 10.1016/S0140-6736(20)30551-1 32151326 PMC7124523

[121] Dikid T Chaudhary S Goel K Padda P Sahu R Kumar T. Responding to COVID-19 pandemic: why a strong health system is required. Indian J Med Res. (2020) 151:140–5. 10.4103/ijmr.IJMR_761_20 32317411 PMC7366545

[122] De Ceukelaire W Bodini C. We need strong public health care to contain the global corona pandemic. Int J Heal Serv. (2020) 50:276–7. 10.1177/0020731420916725 32188308 PMC7140782

[123] Miles D Stedman M Heald A. Living with COVID-19: balancing costs against benefits in the face of the virus. Natl Inst Econ Rev. (2020) 253:R60–76. 10.1017/nie.2020.30

[124] Patton S. The peace of westphalia and it affects on international relations, diplomacy and foreign policy. Hist. (2019) 10:5. Available online at: https://digitalcommons.lasalle.edu/the_histories/vol10/iss1/5

[125] Kissinger H. World Order: Reflections on the Character of Nations and the Course of History. New York NY: Penguin Pr. (2014).

[126] The Lancet. Reviving the US CDC. Lancet. (2020) 395:1521. 10.1016/S0140-6736(20)31140-5 32416772 PMC7255307

[127] Kim YC Dema B Reyes-Sandoval A. COVID-19 vaccines: breaking record times to first-in-human trials. NPJ Vaccines. (2020) 5:19–21. 10.1038/s41541-020-0188-3 32377399 PMC7193619

[128] Mallapaty BS Callaway E Kozlov M Ledford H Noorden R Van. How COVID vaccines shaped 2021 in eight powerful charts. Nature. (2021) 600:580–583. 10.1038/d41586-021-03686-x 34916666

[129] Cohn BA Cirillo PM Murphy CC Krigbaum NY Wallace AW. SARS-CoV-2 vaccine protection and deaths among US veterans during 2021. Science. (2021) 375:331-336. 10.1126/science.abm0620 34735261 PMC9836205

[130] Zhang Y Banga Ndzouboukou J-L Gan M Lin X Fan X. Immune evasive effects of SARS-CoV-2 variants to COVID-19 emergency used vaccines. Front Immunol. (2021) 12:1–14. 10.3389/fimmu.2021.771242 34880867 PMC8645832

[131] Markandya A Sampedro J Smith SJ Van Dingenen R Pizarro-Irizar C Arto I. Health co-benefits from air pollution and mitigation costs of the Paris agreement: a modelling study. Lancet Planet Heal. (2018) 2:e126–33. 10.1016/S2542-5196(18)30029-9 29615227

[132] Dobson AP Pimm SL Hannah L Kaufman L Ahumada JA Ando AW. Ecology and economics for pandemic prevention. Science. (2020) 369:379–81. 10.1126/science.abc3189 32703868

[133] Pereira M Oliveira AM. Poverty and food insecurity may increase as the threat of COVID-19 spreads. Public Health Nutr. (2020) 23:3236–40. 10.1017/S1368980020003493 32895072 PMC7520649

[134] Dasgupta S van Maanen N Gosling SN Piontek F Otto C Schleussner CF. Effects of climate change on combined labour productivity and supply: an empirical, multi-model study. Lancet Planet Heal. (2021) 5:e455–65. 10.1016/S2542-5196(21)00170-4 34245716

[135] Hsiang S Kopp R Jina A Rising J Delgado M Mohan S. Estimating economic damage from climate change in the United States. Science. (2017) 356:1362–9. 10.1126/science.aal4369 28663496

[136] Bowles DC Butler CD Morisetti N. Climate change, conflict and health. J R Soc Med. (2015) 108:390–5. 10.1177/0141076815603234 26432813 PMC4622275

[137] Lenton TM Rockström J Gaffney O Rahmstorf S Richardson K Steffen W. Climate tipping points — too risky to bet against. Nature. (2019) 575:593–5. 10.1038/d41586-019-03595-0 31776487

